# Formononetin Enhances the Tumoricidal Effect of Everolimus in Breast Cancer MDA-MB-468 Cells by Suppressing the mTOR Pathway

**DOI:** 10.1155/2019/9610629

**Published:** 2019-03-17

**Authors:** Qianmei Zhou, Weihong Zhang, Tian Li, Runwei Tang, Chaoran Li, Shuai Yuan, Desheng Fan

**Affiliations:** ^1^Institute of Interdisciplinary Integrative Medicine Research, Shanghai University of Traditional Chinese Medicine, Shanghai 201203, China; ^2^Breast Surgery Department, Baoshan Branch, Shuguang Hospital Affiliated to Shanghai University of Traditional Chinese Medicine, Shanghai 201900, China; ^3^Pathology Department, Baoshan Branch, Shuguang Hospital Affiliated to Shanghai University of Traditional Chinese Medicine, Shanghai 201900, China

## Abstract

**Background:**

Formononetin, an active ingredient isolated from the traditional Chinese medicinal herb* Astragalus membranaceus*, has anticancer and chemoresistance-reducing biological activities. We evaluated the efficacy of formononetin in improving the tumoricidal effect of everolimus by suppressing the mTOR pathway in breast cancer cells.

**Methods:**

Cell survival was assessed using an MTT assay. Apoptosis was detected using flow cytometry. Proteins related to the mTOR pathway were detected and assessed using real-time PCR and Western blot analysis*. Results.* The results showed that formononetin enhances the efficacy of everolimus in suppressing breast cancer cell growth both in vitro and in vivo. The combination of formononetin and everolimus resulted in a 2-fold decrease in tumor volume and a 21.6% decrease in cell survival. The apoptosis ratio in cells treated with formononetin and everolimus increased by 27.9%. Formononetin and everolimus also inhibited the expression of p-mTOR and p-P70S6K and increased the expression of PTEN and p-4EBP-1. Notably, formononetin alone inhibited p-Akt expression but not everolimus.

**Conclusions:**

Formononetin enhances the tumoricidal effect of everolimus by inhibiting the activity of Akt.

## 1. Introduction

Breast cancer is the most common malignant tumor in women [1]. The incidence of breast cancer among women in China is rising [2, 3]. Triple-negative breast cancer (TNBC) is a special type of breast cancer, where expressions of estrogen receptor, progesterone receptor, and human epidermal growth factor receptor-2 (Her-2) are all negative. It accounts for approximately 10%–20% of all breast cancers. It has the worst prognosis among all breast cancer types, with characteristics such as rapid metastasis, drug resistance, and high mortality [4].

mTOR is a type of serine/threonine protein kinase belonging to the PI3k family. It plays an important role in protein synthesis and autophagy. Abnormal expression of mTOR leads to conditions such as diabetes and tumor development [5]. mTOR has 2 key complexes, the mTORC1 and mTORC2. mTORC1 promotes protein synthesis by phosphorylating 2 key effectors, namely, p70S6 kinase 1 (S6K1) and eIF4E binding protein (4EBP) [6]. mTORC1 regulates cell growth and metabolism, whereas mTORC2 regulates cell proliferation and survival by phosphorylating the AGC family of kinases (PKA/PKG/PKC). mTORC2 is critical to the phosphorylation and activation of Akt. The activated Akt promotes cell survival, proliferation, and growth [7, 8].

Everolimus is an inhibitor of serine-threonine kinase mammalian target of rapamycin (mTOR) [9]. It has been reported that everolimus has broad antitumor activities in preclinical models and used in combination with trastuzumab in several clinical trials [10]. However, the effect of everolimus against TNBC does not have satisfactory efficacy for clinical use.

Formononetin is an active ingredient isolated from the traditional Chinese medicinal herb* Astragalus membranaceus* and has various pharmacologic effects, such as tumor growth inhibition, wound healing, estrogen-like effects, antioxidant activity, and anti-inflammatory effects [11, 12]. Formononetin can exert antitumor effects by inducing cell apoptosis, arresting the cell cycle, inhibiting angiogenesis, and reversing multidrug resistance [13, 14].

Recent studies have shown that formononetin can inhibit tumor growth and induce apoptosis by regulating the PI3k pathway [15]. However, whether the combination of formononetin and everolimus can synergistically provoke cancer cell death remains unclear. In this study, we showed that formononetin significantly enhances the tumoricidal effect of everolimus both in vitro and in vivo. Most importantly, we determined the underlying mechanisms for this effect in MDA-MB-468 cells.

## 2. Materials and Methods

### 2.1. Reagents and Cell Culture

Everolimus was purchased from Sigma-Aldrich (MO, USA). Formononetin was obtained from the National Institute for the Control of Pharmaceutical and Biological Products (Beijing, China). Annexin V-FITC and propidium iodide (PI) were obtained from Sigma-Aldrich (MO, USA). The antibodies against mTOR, p-mTOR, Akt, p-Akt, PTEN, p-4EBP-1, and p-p70s6k were obtained from Cell Signaling Technology (MA, USA). Human breast cancer MDA-MB-468 cells were cultured in an RPMI 1640 medium supplemented with 10% fetal calf serum and 0.01 mg/mL insulin at 37°C with 5% CO_2_ in a humidified atmosphere.

### 2.2. Tumor Xenograft and Treatment

Seven-week-old female nu/nu athymic mice, 18-20 g, were obtained from Academia Sinica (Shanghai, China). All procedures conformed to animal welfare considerations and were approved by the Ethical Committee of Shanghai Traditional Chinese Medicine (09001, March 5, 2014). MDA-MB-468 cells (1 × 10^7^/mL) were injected into the mammary fat pad (m.f.p.) of the mice [16]. When tumors developed (approximately 10 days), the mice (n = 10) were treated with formononetin 50 mg/kg [17] and everolimus 2 mg/kg (Animal dosage of everolimus was converted according to clinical dosage) and combined treatment of formononetin (50 mg/kg) and everolimus (2 mg/kg). Untreated animals were given physiological saline as control. All mice were treated once a day via gavage for 4 weeks. After 4 weeks of treatment, blood was collected from the eyes and animals were sacrificed by cervical dislocation. The tumors were immediately removed, freed from connective and adipose tissue, and weighed.

### 2.3. Cell Growth Inhibition Test

The cell survival was determined by 3-(4,5-dimethylthiazol-2-yl)-2,5-diphenyltetrazolium bromide (MTT) assay [18]. Various concentrations of formononetin with or without everolimus were then added to the MDA-MB-468 cells for varying lengths of time followed by the addition of MTT for another 4 h. Cytotoxicity was expressed as a percentage of (number of cells surviving/total number of untreated cells).

### 2.4. Flow Cytometric Analysis

MDA-MB-468 cells (10^6^/mL) were cultured in 6-well plates. When the culture reached 70%–80% confluence, cells were treated with formononetin (150 *μ*mol/L), everolimus (100 nmol/L), or formononetin (150 *μ*mol/L) plus everolimus (100 nmol/L) for 48 h. Cells were subjected to annexin V-PI dual staining assay or PI staining according to the manufacturer's protocol. Stained cells were identified using a fluorescence-activated cell sorter (Becton Dickinson, CA, USA), and the percentage of apoptotic cell population was determined using ModFit LT3.0 software (Becton Dickinson, CA, USA).

### 2.5. RNA Isolation, Reverse Transcription, and Real-Time PCR

Equal amounts (2 *μ*g) of total mRNA from formononetin and everolimus-treated cells were subsequently transcribed into cDNA using M-MuLV reverse transcriptase (Thermo Fisher Scientific) (Waltham, MA, USA). All reactions were performed in a final volume of 20 *μ*L. The qRT-PCR reaction conditions were as follows: activation at 95°C for 10 min with 40 cycles of denaturation at 95°C for 15 s, primer annealing and extension at 60°C for 1 min, and ramping back to 95°C. Human-specific primers for 5′-GCAATATGTTCATAACGATGGCTGTGG-3′ (PTEN forward) and 5′-GAACTGGCAGGTAGAAGGCAACTC-3′ (PTEN reverse), 5′-TTGGAGAACCAGCCCATAAGA-3′ (mTOR forward) and 5′-ATGAGATGTCGCTTGCTTGATAA-3′ (mTOR reverse), 5′-CGCCTGCCCTTCTACAACC-3′ (Akt forward) and 5′-TCATACACATCTTGCCACACGA-3′ (Akt reverse), 5′-GGGCTAGCGATGTCCGGGGGCAGCAGCTG-3′ (4EBP1 forward) and 5′-GGAAGCTTAATGTCCATCTCAAACTGTGACTC-3′ (4EBP1 reverse), 5′-GGGCTAGCGATGAGGCGACGAAGGAGGCGG-3′ (p70s6k forward) and 5′-GGGGTACCTAGATTCATACGCAGGTGCTCTG-3′ (p70s6k reverse) were designed. Expression was assessed using the ΔCt method.

### 2.6. Western Blot Analysis

When human breast cancer MDA-MB-468 cells in 6-well plates reached 90% confluence, the cells were washed with PBS and cell total proteins were extracted. Then proteins were subjected to SDS-PAGE and Western blot analysis. Protein expressions were detected using primary antibodies (1:1000) and secondary antibodies (1:800) conjugated with horseradish peroxidase and ECL reagents (Pharmacia, Buckinghamshire, UK). Quantitative analyses of Western blots were performed using Alpha Ease FC (FluorChem FC2) software. The density ratio of proteins to GAPDH as spot density was calculated using analysis tools.

### 2.7. siRNA Transfection

Transfection was performed with Lipofectamine 2000 (Invitrogen, California, USA) following the manufacturer's instructions. siRNA transfection was performed 24 h before formononetin and everolimus treatment. siRNA duplexes, including Akt siRNA (sc-43609) and control-scrambled siRNA (sc-37007), were obtained from Santa Cruz Biotechnology (Dallas, TX, USA).

### 2.8. Statistical Analysis

Statistical differences were identified using 2-tailed Student t tests. Data are presented as mean ± standard deviation. A p value of < 0.05 was considered statistically significant.

## 3. Results

### 3.1. Combined Formononetin and Everolimus Treatment Suppresses Tumor Masses Significantly

**Figure 1:**
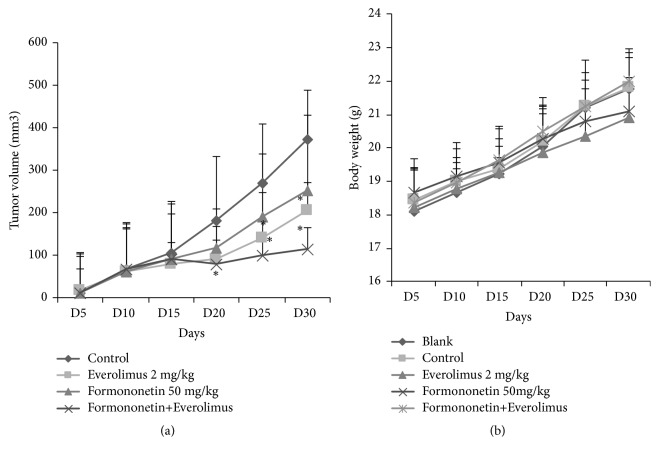
Effect of formononetin or formononetin plus everolimus on tumor outgrowth. (a) Mice were first injected with MDA-MB-468 cells for 2 weeks to establish the xenograft model. Mice were then administered formononetin (50 mg/kg), everolimus (2 mg/kg), or formononetin plus everolimus. After 4 weeks, mice were sacrificed and tumors were excised and weighed. *∗*p < 0.05 versus control. (b) Body weights of animals treated with formononetin or formononetin plus everolimus for 4 weeks.

To determine the effect of formononetin on in vivo tumor outgrowth, we used MDA-MB-468 breast cancer xenografts. Tumor growth was significantly inhibited in the formononetin 50 mg/kg alone group compared with the control group (p < 0.05). Tumor volume shrank from 472.7 to 253.6 mm^3^ on the 30th day of tumor growth. Moreover, in the presence of formononetin, everolimus resulted in a 2-fold reduction in tumor volume. These results suggest that formononetin can synergistically enhance the tumoricidal effect of everolimus in human breast cancer cells (Figure 1(a)).

Furthermore, formononetin was observed to be safe in the MDA-MB-468 xenograft model. None of the mice died with everolimus alone or combined formononetin-everolimus treatment. Mice receiving formononetin and everolimus had no apparent weight loss and had a healthy appetite (Figure 1(b)). These results suggest that formononetin inhibited tumor growth safely.

### 3.2. Formononetin Significantly Enhances the Tumoricidal Effect of Everolimus

**Figure 2:**
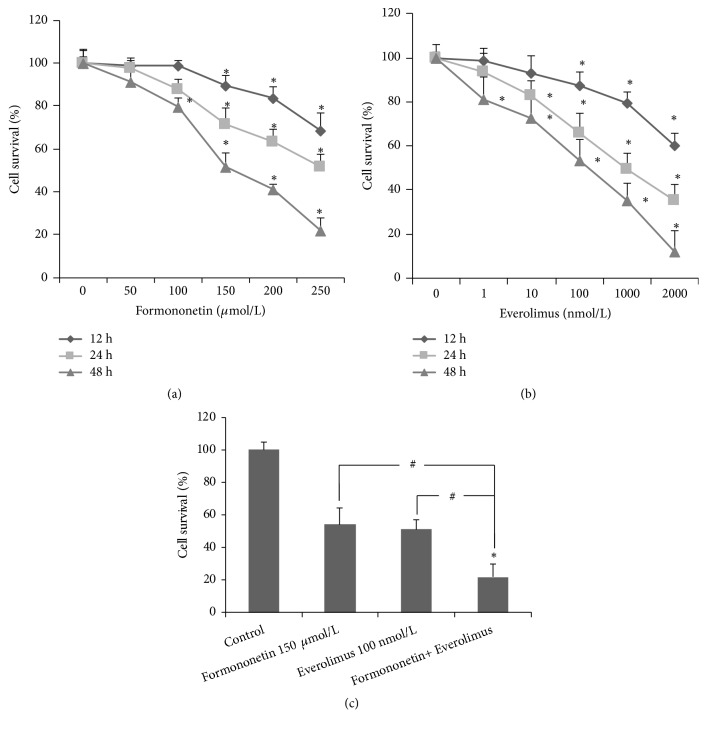
Sensitivities of MDA-MB-468 cells to formononetin and everolimus. An MTT assay was performed to determine the cell number, as described in Section 2. MDA-MB-468 cells were incubated in formononetin at 50, 100, 150, 200, and 250 *µ*mol/L (a) and everolimus at 1, 10, 100, 1000, and 2000 nmol/L (b) for 12, 24, and 48 h. (c) Both everolimus alone or formononetin and everolimus combined inhibited cell growth for 48 h. Values are means ± SE from 3 independent experiments. *∗*p < 0.05 versus control. ^#^p<0.05 versus both everolimus and formononetin alone.

To determine the underlying mechanism, we analyzed the cytotoxicity of formononetin with or without everolimus on MDA-MB-468 cells. Cells were then exposed to various concentrations of formononetin and everolimus for 12, 24, and 48 h. Cell viability was determined using an MTT assay. The results showed that formononetin or everolimus alone inhibited cell survival in a dose- and time-dependent manner. Moreover, the half-maximal inhibitory concentrations of formononetin and everolimus alone were 150 *µ*mol/L and 100 nmol/L for 48 h, respectively (Figures 2(a) and 2(b)). When formononetin was used with everolimus, cell survival decreased by 21.6%. These results suggest that formononetin alone can inhibit breast cancer cell growth. Formononetin can synergistically enhance the tumoricidal effect of everolimus in MDA-MB-468 breast cancer cells (Figure 2(c)).

### 3.3. Formononetin and Everolimus Synergistically Induce Apoptosis in MDA-MB-468 Cells

**Figure 3:**
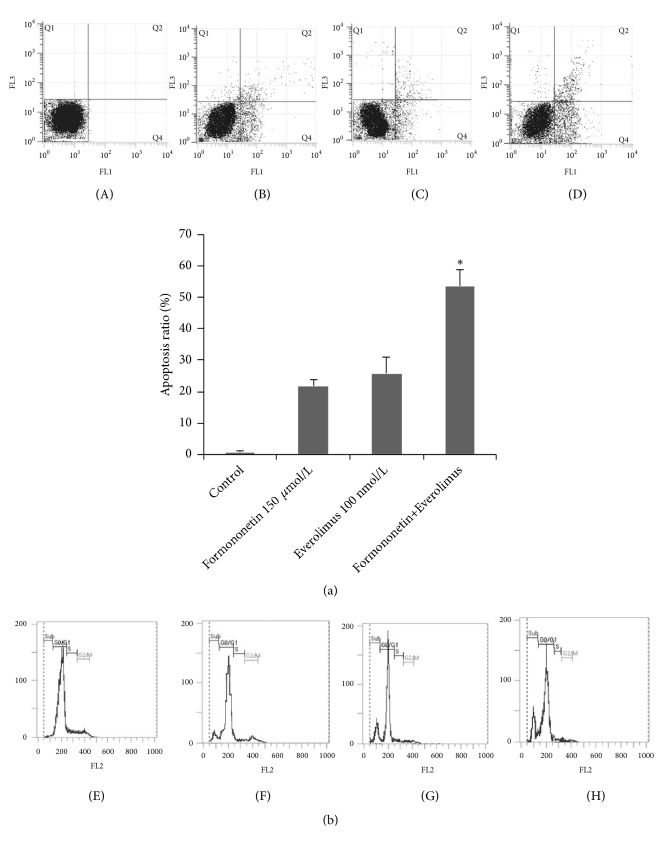
Effect of everolimus alone or both formononetin and everolimus on apoptosis in MDA-MB-468 cells. MDA-MB-468 cells were treated with formononetin (150 *µ*mol/L), everolimus (100 nmol/L), or both for 48 h. Cells were subsequently stained for annexin V-PI (a) and PI only (b) followed by flow cytometric analysis. (A) and (E) control, (B) and (F) formononetin (150 *µ*mol/L), (C) and (G) everolimus (100 nmol/L), and (D) and (H) formononetin + everolimus. *∗*p<0.05 versus formononetin or everolimus alone.

To determine the effect of formononetin and everolimus treatment on cell apoptosis, we performed flow cytometry on MDA-MB-468 cells that were exposed to either one of the drugs or both for 48 h. The apoptosis ratio among cells treated with formononetin (150 *µ*mol/L), everolimus (100 nmol/L), or formononetin + everolimus was 21.5%, 25.7%, and 53.8%, respectively (Figure 3(a)). Cell cycle analysis showed that hypodiploid peaks also appeared with different treatments (Figure 3(b)). These results indicate that formononetin and everolimus result in apoptosis.

### 3.4. Effect of Formononetin and Everolimus on the mTOR Pathway

**Figure 4:**
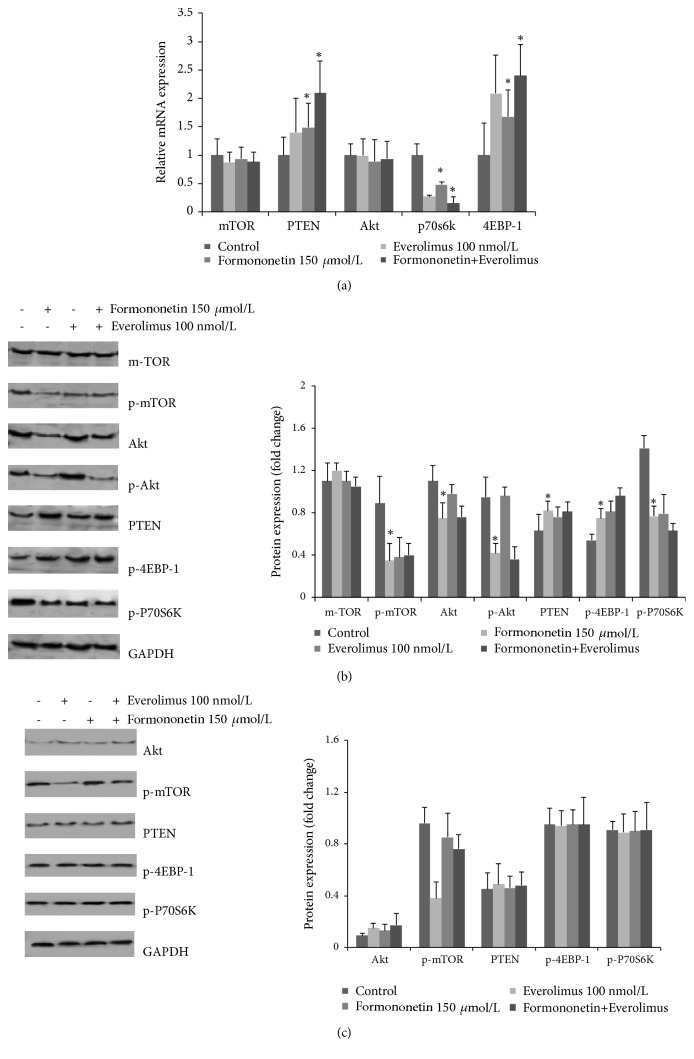
Effect of everolimus alone or both formononetin and everolimus on the mTOR pathway. MDA-MB-468 cells were treated with formononetin (150 *µ*mol/L), everolimus (100 nmol/L), or both for 48 h. (a) PTEN, mTOR, Akt, P70S6K, and 4EBP-1 mRNA were identified using qRT-PCR analysis.*∗*p<0.05 versus control. (b) The expressions of mTOR, p-mTOR, Akt, p-Akt, PTEN, p-4EBP-1, and p-P70S6K were determined using Western blotting. (c) In the presence of Akt siRNA, the levels of p-mTOR, Akt, PTEN, p-4EBP-1, and p-P70S6K were observed on treatment with either everolimus alone or both formononetin and everolimus.

Tumor growth has been shown to be regulated through the mTOR pathway. Everolimus is an inhibitor of mTORC1. In this study, we investigated the mechanisms by which formononetin exerted its effect on tumor growth using qRT-PCR. PTEN mRNA and 4EBP-1 mRNA had higher expression in the formononetin group than in the control group. P70s6k mRNA levels decreased (p<0.05) (Figure 4(a)). We further confirmed the efficacy of formononetin through Western blotting. Formononetin and everolimus also inhibited the expression of p-mTOR and p-P70S6K and increased that of PTEN and p-4EBP-1. However, formononetin alone inhibited the level of p-Akt but everolimus did not (Figure 4(b)).

To evaluate the effect of formononetin on the Akt pathway, silencing of Akt was validated using Western blotting. We found that the level of mTOR was restored to that in the control group after application of formononetin. However, in the presence of Akt siRNA, everolimus had no significant effect on the expression of mTOR. The expressions of p-4EBP-1 and p-P70S6K were all reversed by formononetin with Akt siRNA (Figure 4(c)). These results demonstrate that the inhibition of the mTOR pathway by formononetin is associated with Akt.

## 4. Discussion

mTOR is often considered a downstream effector of numerous mutant oncogene pathways, such as the PI3K/Akt pathway and the Ras/Raf/Mek/Erk (MAPK) pathway, causing overactivation of mTOR and hence cancer [19]. Patients with overexpression of p-mTOR had worse prognosis in early tri-negative breast cancer [20]. Everolimus was found to have inhibitory activity only on the mTORC1 complex and had no apparent effect on the mTORC2 complex [21]. This indicates its limitations as an antitumor agent. In this study, we evaluated whether formononetin can inhibit tumor growth by suppressing the mTOR pathway and whether it can enhance the efficacy of everolimus.

We found that formononetin alone could inhibit the growth of breast cancer cells; our results are in line with those of a previous study [14]. We further showed that formononetin improved the efficacy of everolimus in suppressing breast cancer cell growth both in vitro and in vivo (Figures 1 and 2). In this study, we showed that a clinical dosage of 2 mg/kg everolimus significantly decreased tumor masses in the MDA-MB-468 xenograft model. Notably, the inclusion of formononetin in everolimus treatment resulted in a 2-fold decrease in tumor volume compared with that with everolimus alone (Figure 1(a)). Moreover, the combination treatment is safe (Figure 1(b)). Our results strongly indicate that formononetin may be used to enhance antitumoricidal effect of everolimus.

Proliferation and apoptosis of tumor cells are the key steps in the onset and development of cancer [22]. We found that both formononetin and everolimus or everolimus alone inhibited cell growth in MDA-MB-468 cells (Figure 2). To determine whether cell death induced by formononetin and everolimus was related to apoptosis, the apoptosis rate and cell cycle were evaluated. The results showed that the combination of formononetin and everolimus results in a 2-fold increase in apoptosis (Figure 3). Our results support the hypothesis that the synergistic tumor-killing effect of formononetin and everolimus treatment at least partially contributes to a greater efficacy in inducing apoptosis than with formononetin or everolimus alone.

Moreover, we found that formononetin and everolimus alone inhibited the expression of p-mTOR and p-P70S6K and increased that of p-4EBP-1. However, formononetin alone inhibited the level of p-Akt and everolimus did not (Figures 4(a) and 4(b)). In the presence of Akt siRNA, the expressions of p-4EBP-1 and p-P70S6K were all reversed by formononetin. These results suggest that the inhibition of the mTOR pathway by formononetin is associated with Akt. Phosphorylation and activation of Akt are the most critical roles of mTORC2 [23, 24]. Therefore, we conclude that formononetin may enhance the antitumor effect of everolimus by additionally inhibiting mTORC2.

In summary, our study demonstrated that formononetin can improve the tumoricidal effect of everolimus. Formononetin can augment everolimus in inhibiting the mTOR pathway by effectively inhibiting mTORC2. The combination treatment of formononetin and everolimus may be an effective approach for breast cancer chemotherapy.

## Data Availability

The data used to support the findings of this study are available from the corresponding author upon request.
